# Long-Term Fish Oil Supplementation Induces Cardiac Electrical Remodeling by Changing Channel Protein Expression in the Rabbit Model

**DOI:** 10.1371/journal.pone.0010140

**Published:** 2010-04-13

**Authors:** Xulin Xu, Min Jiang, Yuhong Wang, Timothy Smith, Clive M. Baumgarten, Mark A. Wood, Gea-Ny Tseng

**Affiliations:** 1 Department of Physiology and Biophysics, Virginia Commonwealth University, Richmond, Virginia, United States of America; 2 Department of Chemistry, University of Richmond, Richmond, Virginia, United States of America; 3 Division of Cardiology, Department of Internal Medicine, Medical College of Virginia, Richmond, Virginia, United States of America; University of Toronto, Canada

## Abstract

Clinical trials and epidemiological studies have suggested that dietary fish oil (FO) supplementation can provide an anti-arrhythmic benefit in some patient populations. The underlying mechanisms are not entirely clear. We wanted to understand how FO supplementation (for 4 weeks) affected the action potential configuration/duration of ventricular myocytes, and the ionic mechanism(s)/molecular basis for these effects. The experiments were conducted on adult rabbits, a widely used animal model for cardiac electrophysiology and pathophysiology. We used gas chromatography - mass spectroscopy to confirm that FO feeding produced a marked increase in the content of n-3 polyunsaturated fatty acids in the phospholipids of rabbit hearts. Left ventricular myocytes were used in current and voltage clamp experiments to monitor action potentials and ionic currents, respectively. Action potentials of myocytes from FO-fed rabbits exhibited much more positive plateau voltages and prolonged durations. These changes could be explained by an increase in the L-type Ca current (I_CaL_) and a decrease in the transient outward current (I_to_) in these myocytes. FO feeding did not change the delayed rectifier or inward rectifier current. Immunoblot experiments showed that the FO-feeding induced changes in I_CaL_ and I_to_ were associated with corresponding changes in the protein levels of major pore-forming subunits of these channels: increase in Cav1.2 and decrease in Kv4.2 and Kv1.4. There was no change in other channel subunits (Cav1.1, Kv4.3, KChIP2, and ERG1). We conclude that long-term fish oil supplementation can impact on cardiac electrical activity at least partially by changing channel subunit expression in cardiac myocytes.

## Introduction

Clinical trials and epidemiological studies have suggested that dietary fish oil (FO) supplementation can provide an anti-arrhythmic benefit in some patient populations [1]. One of the largest trials, the GISSI Prevenzione trial, showed that patients that survived recent (<3 months) myocardial infarction when receiving FO supplementation had a reduced mortality rate [2]. There was no reduction in the risk for non-fatal myocardial infarction. The reduced mortality could be attributed, at least partly, to a protection against sudden cardiac death by the FO supplementation [2].

The mechanism(s) underlying the anti-arrhythmic effect of FO supplementation has been under investigation for years. It has been proposed that this anti-arrhythmic effect is mainly due to a direct suppression of Na (I_Na_) and L-type Ca (I_CaL_) currents in cardiac myocytes by the active ingredients of FO, n−3 polyunsaturated fatty acids (PUFAs), such as docosahexaenoic acid (DHA or C22:6,n−3) and eicosapentaenoic acid (EPA or C20:5,n−3) [3]. This is similar to a combination of class I and class IV anti-arrhythmic mechanisms. There are several issues with this proposed anti-arrhythmic mechanism for fish oil or n-3 PUFAs. First, the acute current-suppressing effects observed in tissue bath experiments cannot explain why clinically it takes ∼3 months for FO supplementation to manifest the protective effect [2]. Second, n−6 PUFAs (i.e. arachidonic acid) have similar current-suppressing effects in tissue bath experiments [4], [5]; yet they do not provide anti-arrhythmic protection. Third, although acute exposure to DHA or EPA can suppress I_Na_ in neonatal rat cardiomyocytes [4] or in heterologous expression systems [6], experiments of feeding adult animals with an FO-rich diet for weeks have not shown any I_Na_ reduction [7].

To more closely mimic the clinical situation, it is important to study the effects of dietary FO supplementation in animal models after long-term (weeks to months) FO feeding. To gain insights into the ionic mechanisms for the anti-arrhythmic effects of FO supplementation, it is necessary to study how such treatment can impact on ion channels that are involved in shaping the action potential configuration and duration in the heart. Since clinically the protective effects of FO supplementation lag behind the beginning of FO regimen by about 3 months [2], the involvement of changes in gene expression must be taken into consideration. Therefore, to provide a molecular basis for such changes in ion channel function, it is necessary to examine the expression level of relevant ion channel subunit proteins in cardiac myocytes.

Our goals were to understand: (a) how FO feeding for 4 weeks could affect the action potential configuration and duration in ventricular myocytes, (b) how FO feeding affected the function of ion channels that are important determinants of action potential properties, and (c) whether changes in ion channel function involved alterations in gene expression. We chose a popular animal model, rabbit, in our experiments. Rabbits have been widely used to study cardiac electrophysiology, pathology and pharmacology. Our data confirm that FO feeding induces an ‘electrical remodeling’ in rabbit ventricular myocytes by altering channel gene expression.

## Materials and Methods

### 1. Animal preparation

The investigation conforms with the *Guide for the Care and Use of Laboratory Animals* published by the US National Institutes of Health (NIH Publication No. 85-23, revised 1996). The animal protocol is reviewed by IACUC of VCU annually (IACUC protocol # 10294). Twenty-three young adult (2–2.5 months) male New Zealand White rabbits were included in this study. Ten (FO group) were fed complete content of FO soft gel (NatureMade, ∼120 mg/kg/d of DHA+EPA) for 4 weeks. The others (control group) were not given any fat supplementation. FO-fed rabbits did not show any signs of pathology. Their weight gain during 4-week FO feeding was the same as control rabbits during the same period: at the end of 4 weeks, body weights of control rabbits increased from 2.36±0.05 to 2.90±0.09 kg, while those of FO-fed rabbits increased from 2.38±0.07 to 2.88±0.05 kg (p>0.05). Furthermore, the FO-fed rabbits did not show any signs of myocardial hypertrophy: mean values of cell capacitance were160±15 and 159±6 pF for control and FO myocytes (n = 20 and 33, p>0.05).

For myocyte isolation, the aorta was cannulated and the heart was mounted on a Langendorff apparatus for enzyme treatment (see below). If the heart was used for biochemical experiments, it was dissected into different regions of ∼5×5 mm chunks, snap frozen in liquid nitrogen and stored at −80°C until experiments.

### 2. Single myocyte preparation

The heart was perfused retrogradely through the aorta sequentially with the following oxygenated solutions warmed to 37°C: (1) normal Tyrode's (composition given below), 4–5 min, to monitor the regularity/strength of heart beats, (2) nominally Ca-free Tyrode's supplemented with 0.1% BSA, 6–7 min, to wash out Ca, (3) same solution as (2) but with collagenase (Worthington type II, 0.5 mg/ml), 30 min, to digest the extracellular matrix, and (4) KB solution, 3 min, to stop enzyme action. LV base was minced and the tissue was very gently shaken in KB to release single myocytes. The cell suspension was filtered through a 500-um nylon mesh and stored at room temperature in KB. Experiments were done in ≤8 hr after cell isolation.

### 3. Patch clamp experiments

Cell suspension was added to a poly-lysine-coated coverslip placed in the bath mounted on an inverted Nikon microscope. After allowing cell attachment for 3–5 min, cells were superfused with normal Tyrode's solution at 34±1°C. We used pipettes with tip resistance ∼1–2 MΩ. Patch clamp recordings of whole cell currents were controlled by pClamp 10 via Digidata 1440A, using an Axopatch 200B amplifier. Series resistance was compensated up to 95%. Pipette tip potential was zeroed before making seal, and a liquid junction potential of 10 mV between the pipette and bath solutions (pipette side negative) was corrected during data analysis. Currents were low-pass filtered at 1 kHz (Frequency Devices) and stored for off-line analysis.

### 4. Solutions and drugs

The normal Tyrode's solution contained (mM): NaCl 147, KCl 4, CaCl_2_ 2, MgCl_2_ 0.5, HEPES 5, dextrose 5.5, pH 7.3 (∼295 mOsm). The Na- and Ca-free Tyrode's solution was made by equimolar substitution of NaCl and CaCl_2_ with choline-Cl and MgCl_2_, respectively, and 25 mM sucrose was added to maintain the osmolarity (∼302 mOsm). The pipette solution contained (mM): K-aspartate 110, KCl 20, ATP (K salt) 5, HEPES 5, MgCl_2_ 1, pH 7.3 (∼300 mOsm). The KB solution contained (mM): K-glutamate 110, KH_2_PO_4_ 10, MgSO_4_ 1.8, EGTA 0.5, taurine 10, HEPES 10, and glucose 20 (∼305 mOsm).

DHA (Sigma) was aliquoted in 10 ul volume and stored at −80°C. On each experimental day, 1 ul of DHA was dissolved in 282 ul DMSO to make 10 mM stock solution. The stock solution was kept on ice, and diluted to 10 uM in bath solution right before applying to cells. DHA aliquot and stock solution were discarded at the end of each experimental day. Dofetilide was dissolved in water (pH 3) at 1 mM, aliquoted and stored at −20°C. It was diluted to 1 µM in bath solution right before applying to cells.

### 5. Fatty acid analysis

Lipids were extracted from tissue samples using a procedure modified from that of Folch [8]. All solvents contained butylated hydroxytoluene (BHT, 50 ug/ml) to prevent lipid oxidation. Briefly, tissue (0.3–0.7 g) were minced in methanol (6 ml) and homogenized with polytron grinder for 2 min. Chloroform (12 ml) was added and the mixture was further homogenized for 2 min. Then 0.88% (w/v) KCl 4.5 ml was added and the mixture was vortexed vigorously for 2 min. After the phases separated, the lower organic phase was further extracted with 5 ml of methanol plus 0.88% KCl (1∶1, v/v). The final organic phase was dried down under a stream of nitrogen, reconstituted in 100 ul chloroform and stored at −80°C.

The phospholipid (PL) component was separated from the other components in the lipid extracts using thin-layer chromatography [9]. Samples along with a PL standard (PL mix from soybean, Supelco) of different dilutions in 10 ul total volume were applied to preparative silica gel G plates (20×20 cm, silica gel thickness  = 0.5 mm). The mobile phase was hexane/diethyl ether/acetic acid (85∶15∶1, by vol). After the mobile phase reached within 1 inch to the top, the plate was dried by baking on a hotplate at ∼120°C for 5 min, sprayed with 2′,7′-dichlorofluorescene (in 95% methanol, 0.1% w/v), and the lipid spots were visualized/quantified under UV (ChemiImager model 4400, α-Innotech).

To make the fatty acids volatile at ∼200°C (for separation by gas chromatography), the lipid extracts (25 ul) were transmethylated by boiling in excess (500 ul) boron trifluoride (BF_3_, in methanol 14% w/v) in a boiling water bath for 2–3 min. Methyl esters of long-chain fatty acids were extracted using 1.5 ml hexane/water (3∶2, v/v). The organic phase was dried down under a stream of nitrogen, reconstituted in 25 ul of hexane and stored at −80°C.

The above methylated fatty acid samples were analyzed by gas chromatography - mass spectroscopy (GC-MS, Shimadzu, model QP5050A). We used a GC capillary column (Omegawax250, Supelco) designed for the separation of long-chain polyunsaturated fatty acids. The injection volume was 1 ul with a split ratio of 30:1. The oven temperature was set a 200°C and the carrier gas (helium) was set at a total flow rate of 1 ml/min. The following methylated fatty acid standards (Supelco) were used to confirm peaks identity: C20:4,n–6, C22:6,n–3, C18:1,n–9, and C18:3,n–3.

### 6. Immunoblot analysis

Except for Kv1.4, the protein levels of channel subunits were quantified from membrane-enriched fraction prepared using procedures modified from those described by Takimoto [10]. Frozen tissue chunks were pulverized under liquid nitrogen, and homogenized in 10 vol of buffer (0.25 M sucrose, 1 mM EDTA [pH 7.4]). This and all the following procedures took place in the presence of the protease inhibitor cocktail at 4°C or on ice. The homogenate was centrifuged at 3,500 rpm for 10 min to pellet nuclei and debris. The supernatant was centrifuged at 30,000 rpm for 1 hr to pellet the membranes. The membrane pellet was washed with a solution (Tris-HCl 20 mM [pH 7.4], EDTA 1 mM) and referred to as post-nuclear membrane fraction. The post-nuclear membrane fraction was rehomogenized in a Triton-containing lysis buffer (Tris-HCl 20 mM [pH 7.5], NaCl 0.2 M, EDTA 1 mM, Triton X-100 1%) using Dounce grinder. The mixture was centrifuged at 17,000 rpm for 1 hr, and the supernatant (Triton extract) was used for immunoblotting.

Initial attempts to detect Kv1.4 in the membrane-enriched fraction failed, although the antibody detected a strong ∼100 kDa fuzzy band in whole tissue lysate (WTL) of rat brain (representing glycosylated Kv1.4) and a faint band of a similar size in WTL of rabbit hearts (see below). Therefore, the Kv1.4 data reported here were from WTL prepared using the procedures described by O'Rourke et al [11]. Briefly, frozen tissue chunks were pulverized in 10 vol of lysis buffer (in mM: NaCl 145, MgCl_2_ 0.1, HEPES 15, EGTA 10, pH 7, Triton X-100 0.5, with protease inhibitor cocktail), and solubilized for 30 min on ice. The above was homogenized by tip sonicator (2 of 15-s bursts), and then centrifuged to pellet nuclei and debris. The supernatant was used for immunoblotting. Importantly, the effect of FO feeding on Cav1.2 protein measured in the same set of hearts was similar between these two methods of protein preparation (see below).

The protein concentrations in membrane-enriched fraction of WTL were quantified using BCA kit (Pierce). Protein samples were loaded onto 7.5% or 4–20% gradient SDS polyacrylamide gels. After fractionation, the proteins were blotted to PVDF membranes (Amersham), and probed with the following antibodies: Cav1.2 mAb (NeuroMabs), Cav1.1 mAb (Abcam), Kv4.2 pAb (Sigma), Kv4.3 pAb (Alomone), KChIP2 mAb (NeuroMabs), Kv1.4 mAb (NeuroMabs) and hERG pAb (Alomone). Immunoreactivity was visualized using an ECL detection kit (Amersham), and band intensities were quantified by densitometry (ChemiImager model 4400). The same membranes were stripped and probed for α-actin (Sigma) to check loading variations.

### 7. Data analysis

For patch clamp recordings, the experimental protocol and methods of data analysis are described in text or figure legends. Data were analyzed using Clampfit of pClamp10. Statistical analysis of data from patch clamp experiments, GC-MS analysis, and densitometry was done using SigmaStat (v 2.1). Multiple group data were analyzed by one-way ANOVA and, if p<0.05, followed by pair-wise comparisons. The t-test was used for comparison between two groups. Statistical significance is noted as: *** p<0.001, ** p<0.01, * p<0.05.

## Results

### 1. Fish oil feeding enriches n-3 PUFA content in the phospholipids of rabbit heart

**Figure 1:**
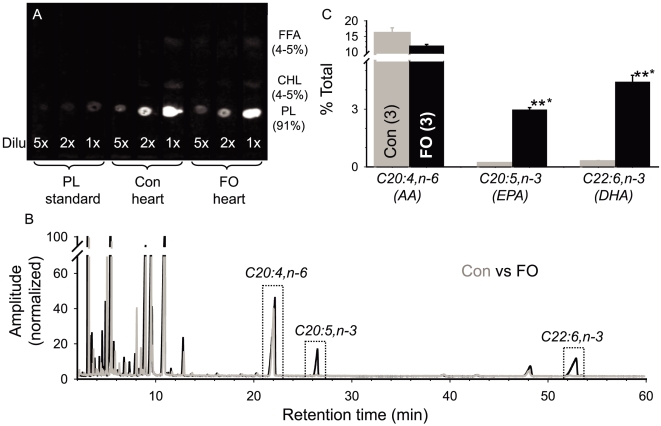
Fish oil (FO) feeding increased the n-3 PUFA content in phospholipids (PLs) of rabbit hearts. Lipids were extracted from ventricular myocardium and analyzed by thin-layer chromatography (TLC), or transmethylated followed by characterization/quantification by gas chromatography-mass spectroscopy (GC-MS). (**A**) Image of a representative TLC plate sprayed with 2′,7′-dichlorofluorescence which made lipid spots fluorescent under UV. The loaded PL standard and lipid samples with their dilutions (Dilu) are labeled on the bottom. The positions of PL, cholesterol (CHL) and free fatty acids (FFA) spots are marked on the right. Spot intensities were quantified by densitometry, and the percentages of the three components are listed. (**B**) Superimposed chromatograms of fatty acids from a control rabbit (gray trace) and an FO-fed rabbit (black trace). Peak amplitudes were normalized by the first peak (butylated hydroxytoluene, antioxidant in solvents at 50 ug/ml). The peaks of interest, C20:4,n−6 (arachidonic acid, AA), C20:5,n−3 (eicosapentaenoic acid, EPA) and C22:6,n−3 (docosahexaenoic acid, DHA) are labeled. (**C**) Data summary. Areas under the peaks of AA, EPA and DHA were normalized by the total areas of peaks above threshold and averaged over three samples from each group.

**Figure 2:**
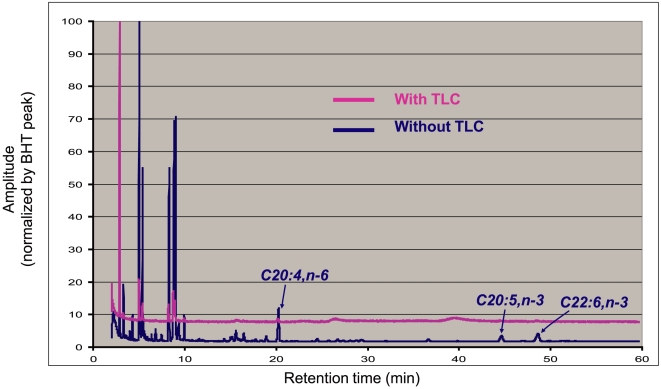
Effects of the thin-layer chromatography (TLC) procedure on downstream lipid analysis. Comparing GC-MS chromatograms of transmethylated lipid samples prepared from the same batch of COS-7 cells, with (magenta) or without (dark blue) the TLC procedure to isolate the phospholipid component in the lipids. Peak amplitudes in the retention time range of <10 min are dramatically reduced after TLC. Importantly, the peaks of long-chain polyunsaturated fatty acids (C20:4,n−6, C20:5,n−3, and C22:6,n−3) are largely missing after TLC, suggesting that the TLC procedures (prolonged exposure to air at elevated temperatures) may have caused extensive oxidation of the polyunsaturated acyl chains, thus invalidating the GC-MS data.

Fig. 1A shows that phospholipids (PLs) accounted for ≥90% of the lipids extracted from both control and FO-fed rabbit hearts. To reduce the risk of oxidation of long-chain polyunsaturated acids, which would invalidate the downstream analysis (as shown in Fig. 2, due to changes in the fatty acid alkyl chain properties), we bypassed the TLC procedure of PL purification and directly subjected lipid extracts to transmethylation and GC-MS analysis.

Fig. 1B shows that we could unequivocally identify C20:4,n−6 (arachidonic acid or AA) and two n−3 PUFA peaks: C20:5,n−3 (eicosapentaenoic acid or EPA) and C22:6,n−3 (docosahexaenoic acid or DHA). FO feeding for 4 weeks markedly increased the content of EPA (from 0.19±0.02 to 2.96±0.12%) and DHA (from 0.28±0.05 to 4.41±0.35%) in rabbit ventricles (Fig. 1C). There was also a slight reduction in AA (from 16.1±1.7 to 12.0±0.5%), although the difference did not reach p<0.05.

### 2. Fish oil feeding elevates the action potential plateau voltage and prolongs the action potential duration of rabbit ventricular myocytes

We measured action potential parameters and the following key plateau phase currents in each of the rabbit ventricular myocytes: transient outward (I_to_), L-type Ca (I_CaL_), delayed rectifier (I_K_), and inward rectifier (I_K1_) currents. To ensure that action potentials and ionic currents were compared among myocytes under similar conditions (i.e., to minimize the issue of current rundown during prolonged whole cell dialysis), after forming the whole-cell recording configuration, all our recordings adhered to the following schedule (time points listed were counted after the formation of whole-cell recording configuration): (1) 0–4 min, allowing whole cell dialysis with the pipette solution and adjusting series resistance compensation, (2) 5–10 min, recording action potentials at 4 cycle lengths (CLs), (3) 10–15 min, recording I_K1_, I_CaL_, and I_to_, (4) 15–20 min, switching the bath solution from normal Tyrode's to Na- and Ca-free Tyrode's while monitoring the disappearance of I_Na_ and I_CaL_ (to remove interference from I_Na_, I_Ca_ and Na/Ca exchanger current, I_NCX_, in the measurement of I_K_), (5) 20–25 min, recording I_K_, (6) 25–30 min, washing in dofetilide 1 uM while monitoring the change in I_K_, and (6) 30–35 min, recording dofetilide-insensitive currents.

Action potentials were elicited by passing suprathreshold 2-ms current pulses via the patch pipette. We tested the effects of FO feeding on action potential configuration and duration at CLs of 0.3, 0.5, 1 and 2 s, to simulate heart rates of bradycardia - tachycardia. For each of the CLs, a train of action potentials was elicited till the configuration and duration reached a steady state (requiring 36–60 action potentials at the CL of 2 s, 72–120 action potentials at the CL of 0.3 s). The order in which the CLs were applied was random among myocytes to avoid the issues of use-dependent changes in the action potential parameters. The last 10 action potentials of a train were averaged and used to measure the resting membrane potential, the action potential plateau height, and the action potential duration.

**Figure 3:**
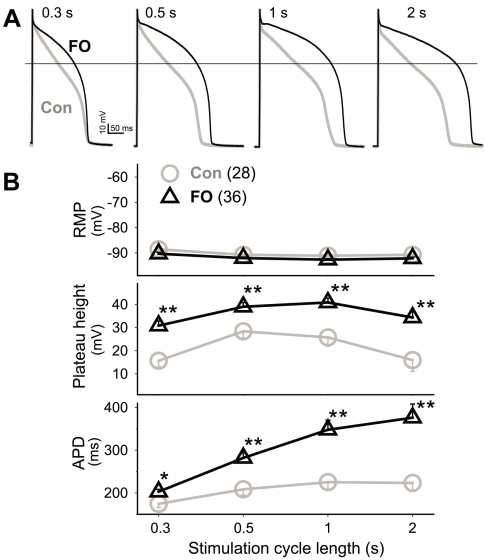
Fish oil feeding elevated action potential plateau height and prolonged action potential duration in rabbit ventricular myocytes. (**A**) Superimposed action potentials recorded from a control (Con) and an FO myocyte at four cycle lengths (marked on top). Horizontal line denotes zero mV. (**B**) Data summary of RMP (resting membrane potential), plateau height (voltage level 50 ms after the upstroke), and APD (action potential duration, measured when membrane was repolarized to −60 mV). Numbers in parentheses are those of myocytes examined.

Fig. 3A depicts representative APs recorded from a control and an FO myocyte, each subjected to stimulation at 4 CLs. Fig. 3B presents data summary. FO feeding markedly elevated the AP plateau height and prolonged APD at all 4 CLs. The degree of APD prolongation was modest at CL 0.3 s, but became more profound at longer CLs. On the other hand, FO feeding did not affect the resting membrane potential.

### 3. Fish oil feeding reduces I_to_ current density and I_to_ subunit expression in rabbit ventricular myocytes

**Figure 4:**
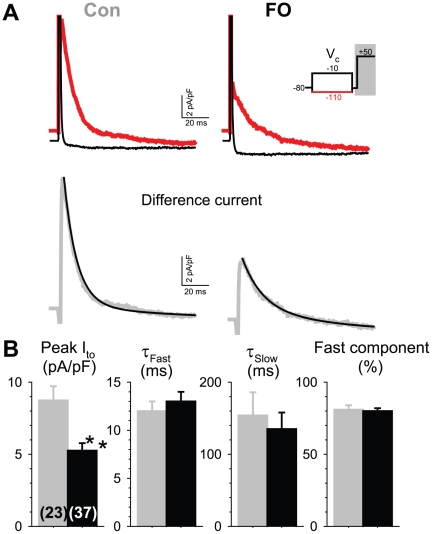
Fish oil feeding caused a decrease in the peak amplitude of transient outward current (I_to_) in rabbit ventricular myocytes without affecting the time course of I_to_ inactivation. (**A**) *Top*: Superimposed current traces from a control and an FO myocyte using the voltage protocol diagrammed in the inset (showing only currents recorded during the shaded area of the protocol). Red and black traces: currents following a conditioning step (V_c_) to −110 and −10 mV, respectively. *Bottom*: Difference currents (gray traces). The difference currents were fit with a 2-exponential function (superimposed black smooth curves). (**B**) Data summary: peak I_to_ density (left, numbers of myocytes studied listed in parentheses), fast and slow time constants of inactivation (τ_Fast_ and τ_Slow_), and % of the fast component at +50 mV.

We quantified the peak amplitude of I_to_ at +50 mV to avoid interference from I_Na_ and I_Ca_, because this voltage was close to the apparent reversal potentials of Na and Ca currents. The voltage clamp protocol is diagrammed in the inset of Fig. 4A. From a holding voltage (V_h_) of −80 mV, a 2-s conditioning pulse to −110 mV was applied to remove I_to_ inactivation [12], so that a fully available I_to_ along with other overlapping currents was recorded during the subsequent test pulse to +50 mV (solid trace). Then a 2-s conditioning pulse to −10 mV was applied to maximally inactivate I_to_ [12], so that a current trace without I_to_ was recorded during the step to +50 mV (dotted trace). The difference current between the two represents fully available, isolated I_to_ (lower traces of Fig. 4A). FO feeding decreased the peak I_to_ current densities from 8.7±1.0 to 5.2±0.5 pA/pF (Fig. 4B, first panel). I_to_ inactivation followed a double exponential time course (double exponential fits in Fig. 4A, lower panel). FO feeding did not change the time course of I_to_ inactivation (Fig. 4B, second to fourth panels).

**Figure 5:**
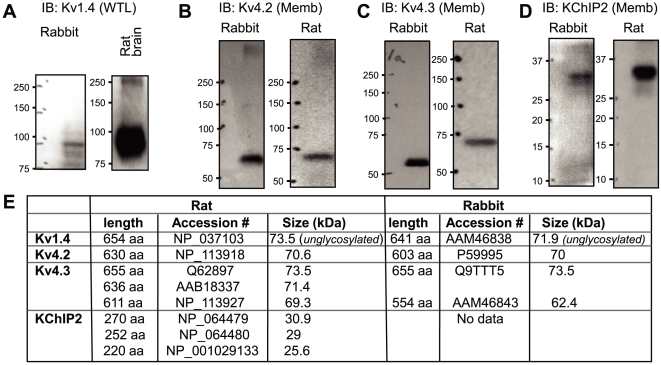
Validation of Abs used to detect channel subunit proteins in the rabbit hearts. (**A**) – (**D**) Immunoblot images of rabbit and rat hearts (except (A), right lane, rat brain), probed with Abs marked on top. Kv1.4 was detected in whole tissue lysates (WTL), while the other three proteins were detected in membrane-enriched fraction (Memb). (**E**) Lengths, accession numbers and molecular sizes (in kDa) of rat and rabbit channel subunit orthologs currently available in the NCBI protein database. The Kv1.4 sizes are those of unglycosylated forms, which are smaller than the N-glycosylated forms shown in (A). Kv4.2 and Kv4.3 do not have N-glycosylation signals. The rabbit Kv4.3 we detected (∼60 kDa, panel (C), left lane) was likely the short isoform (62.4 kDa). KChIP2 is a cytosolic protein, and thus is not glycosylated.

To understand why the I_to_ peak current density was reduced in FO-fed rabbit ventricle, we used immunoblotting to quantify the protein levels of I_to_ channel subunits. Rabbit cardiac I_to_ has 2 components: Kv4.x-based (Kv4.2 and Kv4.3 as pore-forming subunits, KChIP2 as auxiliary subunit) and Kv1.4-based channels [13]. We used Kv4.2, Kv4.3 and Kv1.4 Abs raised against rat sequences, that are 94%, 98% and 100% identical in rabbit sequences. The KChIP2 Ab was also raised against a rat sequence. We could not find information on rabbit KChIP2 in the NCBI database. Fig. 5 shows validation of these Abs as tools to detect target proteins in the rabbit heart, with rat brain or rat heart proteins as positive controls.

**Figure 6:**
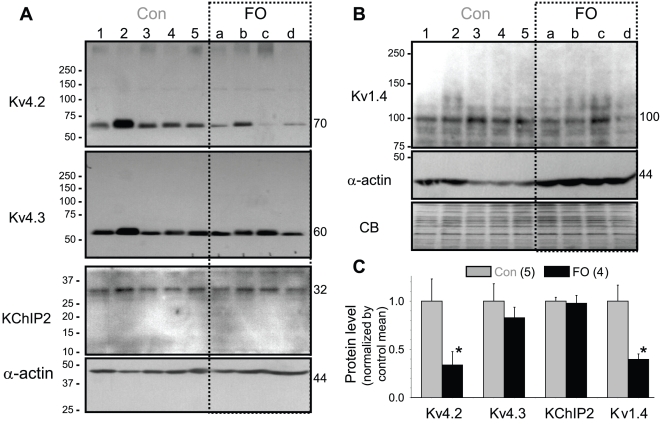
Fish oil feeding caused a decrease in the protein levels of Kv4.2 and Kv1.4, but not Kv4.3 or KChIP2, in rabbit ventricles. (**A**) Immunoblot images of Kv4.2, Kv4.3, and KChIP2 (∼80 ug/lane). (**B**) Immunoblot images of Kv1.4 (∼160 ug/lane). Size marker bands (in kDa) are listed on the left. Sizes for proteins of interest are marked on the right. Note that for panel (A) here and Fig. 8A, each channel subunit immunoblot was corrected by its own α-actin immunoblot for loading variations, although only one representative α-actin immunoblot is shown. Loading variation in Fig. 6B was further checked by coomassie blue (CB) stain. (**C**) Data summary: background-subtracted band intensities were divided by corresponding α-actin band intensity, and then normalized by the mean value of control lanes.

Fig. 6A and 6B depict immunoblot images of I_to_ channel subunits in five control and four FO-fed rabbit hearts. We used α-actin as the internal control to correct for variations in protein loading among lanes. Fig. 6C presents data summary of densitometry quantification. Among the four I_to_ channel subunits examined, Kv4.2 and Kv1.4 protein levels in FO-fed rabbit ventricle dropped to 0.34±0.14 and 0.40±0.05 of control. There was no change in the protein level of Kv4.3 or KChIP2.

### 4. Fish oil feeding increases I_CaL_ current density and I_CaL_ subunit expression in rabbit ventricular myocytes

**Figure 7:**
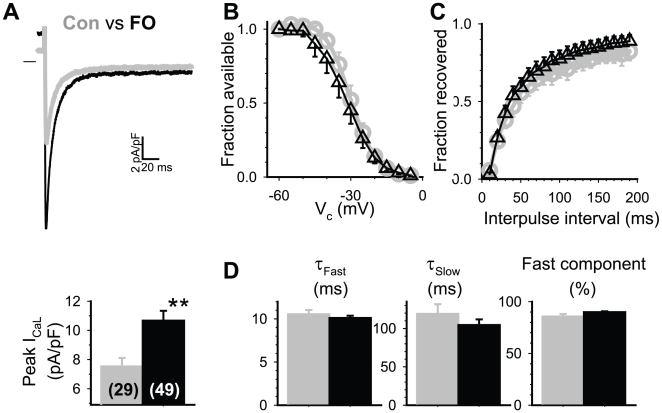
Fish oil feeding caused an increase in the peak amplitude of L-type Ca channel current (I_CaL_) in rabbit ventricular myocytes without altering I_CaL_ gating kinetics. (**A**) *Top*: Superimposed current traces recorded from a control and an FO myocyte. Currents were elicited from a holding voltage (V_h_) of −50 mV to 0 mV. *Bottom*: summary of peak I_CaL_ (numbers of myocytes examined in parentheses). (**B**) Voltage-dependence of inactivation was analyzed using the following protocol. From V_h_ −50 mV, 2-s conditioning pulses to V_c_ −60 to −5 mV in 5 mV steps were applied. After a 5-ms step to −50 mV to reset the capacitive transient, test pulses to 0 mV were used to monitor the availability of I_CaL_. Peak amplitudes of I_CaL_ during the test pulses were normalized by the maximal I_CaL_ following V_c_ to −60 mV, to estimate ‘fraction available’. The relationship between ‘fraction available’ and ‘V_c_’ was fit with a simple Boltzmann function: fraction available  = 1/[1+exp((V_c_−V_0.5_)/k)], where V_0.5_ and k are half-maximum inactivation voltage and slope factor, respectively. The V_0.5_ and k values are (mV): −29.9±2.1 and 4.5±0.3 for control myocytes, −32.1±1.6 and 5.3±0.3 for FO myocytes. (**C**) Time course of recovery from inactivation was analyzed using the following protocol. From V_h_−50 mV, double pulses each to 0 mV for 500 ms with varying interpulse interval (10 to 190 ms) were applied once every 10 s. The peak amplitude of I_CaL_ during the second pulse was normalized by that during the first pulse to estimate ‘fraction recovered’. The relationship between ‘fraction recovered’ and ‘interpulse interval’ was fit with a double exponential function. The percentage and time constant of the fast (major) component are 81±7% and 50.2±4.9 ms for control myocytes, 89±2% and 55.2±6.0 ms for FO myocytes. (**D**) Time course of I_CaL_ inactivation at 0 mV. I_CaL_ at 0 mV was fit with a double exponential function. Shown are fast and slow time constants of inactivation and % of the fast component.

We estimated the peak I_CaL_ by the difference between the inward peak and the current level at the end of a 500 ms pulse from V_h_ −50 mV to 0 mV, where the maximal I_CaL_ occurred. Representative current traces from a control and an FO myocyte are superimposed in the top panel of Fig. 7A. The maximal peak I_CaL_ density was markedly increased by FO feeding (from 7.5±0.6 to 10.7±0.7 pA/pF, Fig. 7A, lower panel). We also characterized the voltage-dependence of I_CaL_ inactivation (Fig. 7B), time course of I_CaL_ recovery from inactivation at −50 mV (Fig. 7C), and the time course of I_CaL_ inactivation during depolarization to 0 mV (Fig. 7D). FO feeding did not induce any detectable changes in these parameters of I_CaL_ gating kinetics.

**Figure 8:**
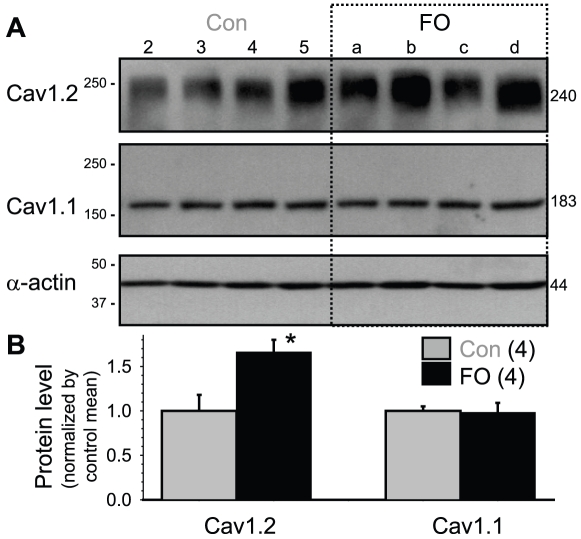
Fish oil feeding caused an increase in the protein level of Cav1.2, but not Cav1.1, in rabbit ventricles. (**A**) Immunoblot images of membrane-enriched fraction from the left ventricular base region of the same set of hearts as shown in Fig. 6 and probed for Cav1.2 and Cav1.1. Loading was ∼80 ug/lane. The membranes were stripped and reprobed for α-actin to check loading. (**B**) Data summary: background-subtracted and loading-corrected band intensities were normalized by the mean value of control lanes.

We used immunoblot experiments to test whether there was a corresponding change in the pore-forming subunit of the L-type Ca channels, Cav1.2. The Cav1.2 Ab was raised against a fusion protein containing partial rabbit Cav1.2 sequence (aa 1507–1733). This Ab recognized a fuzzy band of ∼240 kDa in rabbit ventricles, as expected for glycosylated Cav1.2. FO-feeding caused a significant increase in Cav1.2 protein level in rabbit ventricles (Fig. 8B, 1.66±0.14 vs 1.00±0.18, p<0.05).

Interestingly, a Cav1.1 Ab raised against purified dihydropyridine receptor protein isolated from rabbit skeletal muscle t-tubules could detect a ∼83 kDa sharp band in rabbit ventricles (Fig. 8). There was no change in the Cav1.1 protein level in FO-fed rabbit ventricles, supporting the selectivity of FO-feeding in modulating the Cav1.2 protein level.

**Figure 9:**
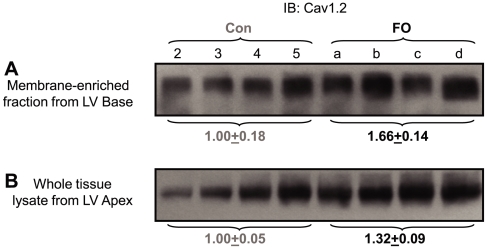
Fish oil (FO)-feeding induced increase in Cav1.2 protein level was similarly seen in both membrane-enriched fraction and in whole tissue lysate prepared from the same set of hearts. The immunoblot image in (A) was the same as that shown in Fig. 8A, top panel. Immunoblot in (B) was from the same PVDF membrane as shown in Fig. 6B, top panel (Kv1.4 immunoblot). The PVDF membrane was stripped of Kv1.4/secondary Abs, and reprobed with the Cav1.2 mAb. Numbers shown below the immunoblots are Mean±SE values of background-subtracted/loading-corrected band intensities normalized by the mean values of control lanes. In both cases, p<0.05, FO vs control (Con).

To check whether the method of sample preparation influenced the results of immunoblot analysis, we compared Cav1.2 quantification in membrane-enriched fraction and in whole-tissue lysate prepared from the same set of hearts. Fig. 9 confirms that immunoblot analysis of both sample preparations reached the same conclusion: FO feeding increased the Cav1.2 protein level in rabbit ventricles.

### 5. Fish oil feeding does not affect delayed or inward rectifier current of rabbit ventricular myocytes

**Figure 10:**
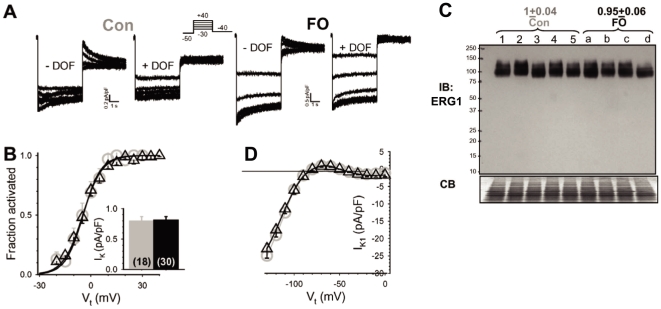
Fish oil feeding did not alter the delayed rectifier (I_K_) or inward rectifier (I_K1_) current in rabbit ventricular myocytes. (**A**) Representative current traces recorded from a control and an FO myocyte, before and during exposure to 1 uM dofetilide (DOF). *Inset*: protocol (V_h_ −50 mV, V_t_ to −30–+40 mV in 5 mV steps for 5 s, V_r_ to −40 mV for 5 s, interpulse interval 30 s). (**B**) Summary of voltage dependence of I_K_ activation (*main graph*) and I_K_ current amplitude (*inset*). The voltage-dependence of activation was analyzed by normalizing the I_K_ tails to the maximal I_K_ tail following V_t_ to +40 mV (fraction activated), and the relationship between ‘fraction activated’ and ‘V_t_’ was fit with a Boltzmann function: fraction activated  = 1/[1+exp((V_0.5_−V_t_)/k)], where V_0.5_ and k are half-maximum activation voltage and slope factor, respectively. The V_0.5_ and k values are (mV): −4.5±2 and 5.2±1.0 for control myocytes, −4.7±1.2 and 5.6±0.4 for FO myocytes. The I_K_ current amplitude was measured from peak tail current following V_t_ to +40 mV (numbers of cells analyzed in parentheses). (**C**) Immunoblot analysis of ERG1 protein level in control and FO rabbit LV (same set of hearts as shown in Figs. 6 and 8). Shown on top are Mean±SE of ERG1-immunoreactive band intensities corrected for loading (divided by CB stain) and normalized by the mean value of control rabbits (p = 0.524). (**D**) Background current-voltage relationship was analyzed using the following voltage clamp protocol. From V_h_ −50 mV, test pulses to V_t_ of 0 to −120 mV in 10 mV steps for 500 ms were applied once every 5 s. Currents were measured at the end of the test pulses.

Fig. 10A shows that in the presence of dofetilide, little or no outward tail current could be detected in either the control or the FO myocyte. Therefore, under our recording conditions the delayed rectifier (I_K_) current in rabbit ventricular myocytes was mainly the rapid component (I_Kr_). FO feeding did not affect the I_K_ current density or the voltage-dependence of I_K_ activation in rabbit ventricular myocytes (Fig. 10B). FO feeding did not alter the protein level of ERG1 (α-subunit of I_Kr_ channels) in rabbit ventricles (Fig. 10C). The background current-voltage relationship in the negative voltage range mainly reflects the inward rectifier (I_K1_) current. Fig. 10D shows that FO feeding did not affect I_K1_ in rabbit ventricular myocytes.

## Discussion

### 1. Molecular mechanisms for fish oil supplementation-induced electrical remodeling

Long-term FO supplementation can affect membrane protein function by at least three mechanisms that are not mutually exclusive. First, incorporation of polyunsaturated acyl chains of n−3 PUFAs into membrane phospholipids will alter the membrane material properties [14]. The increase in membrane fluidity and decrease in lateral pressure may reduce the energy costs of conformational changes in membrane proteins that are critical for their function. Second, an increase in the n−3 PUFA content in the membrane lipid bilayer may facilitate phase separation between PUFA-rich/sphingomyelin and cholesterol-poor disordered lipid domains and PUFA-poor/sphingomyelin and cholesterol-rich ordered lipid domains (lipid rafts) [15]. This can lead to changes in the function and modulation of membrane proteins associated with lipid rafts [16]. Third, PUFAs can modulate gene expression [17] by regulating the activity of transcription factors directly (e.g. sterol regulatory element-binding proteins) or indirectly by binding to nuclear receptors (e.g. peroxisome proliferators-activated receptors) [18]. Our observations that long-term FO feeding altered I_CaL_ and I_to_ channel subunit expression in rabbit hearts suggest that the third mechanism, altered gene expression, is at work.

### 2. Chronic effects of fish oil supplementation vs acute effects of n-3 PUFAs

**Figure 11:**
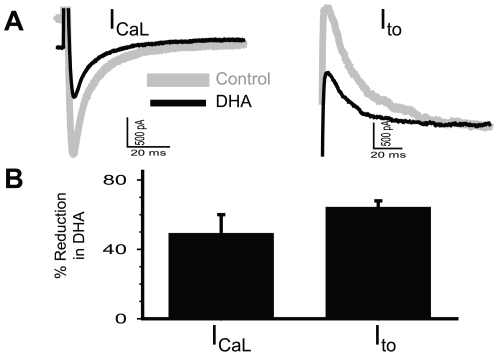
Effects of acute exposure to DHA (10 uM) on I_CaL_ and I_to_ in rabbit ventricular myocytes. I_CaL_ and I_to_ were measured as described for Fig. 7A and Fig. 4A, respectively, before and during exposure to DHA. (**A**) Superimposed current traces. (**B**) Summary of % reduction of peak I_CaL_ and I_to_ in DHA.

Fig. 11 shows that acute application of n−3 PUFA (DHA, 10 µM) markedly suppressed the peak amplitudes of I_CaL_ and I_to_ in rabbit ventricular myocytes. These observations are similar to previous reports of tissue bath experiments [5], [19]. Acute effects of n−3 PUFAs on membrane channels in the heart are likely to occur in vivo transiently after ingestion of FO, when the plasma level of n−3 PUFAs is high. Importantly, the effects of chronic FO feeding on I_CaL_ (enhancement) differ from that of acute DHA application (suppression), while both treatments similarly suppress the I_to_ amplitude. Based on these observations, we suggest that dietary FO supplementation in humans will have both chronic (sustained) and acute (transient) effects, and the two aspects may antagonize or complement each other.

### 3. Clinical Implications

We show that ventricular myocytes isolated from FO-fed rabbits, relative to myocytes from control rabbits, had much more positive plateau heights and longer action potential durations tested at cycle lengths of 0.3–2 s. Our observations were consistent with a previous report showing that FO feeding in rabbits for 30 days caused a prolongation of the plateau phase of monophasic action potentials recorded from the ventricles of Langendorff-perfused hearts [20].

Our observations are different from a previous study in the pig model [7]. FO feeding in pigs caused a decrease in action potential plateau height and a shortening of APD. These changes were accounted for by a decrease in I_CaL_ and I_NCX_, along with an increase in the slow delayed rectifier (I_Ks_) and I_K1_ [7]. The differences in FO feeding-induced cardiac electrical remodeling between rabbit and pig models may be due to a combination of factors: differences in cellular and membrane environment of ion channels in cardiac myocytes which can impact the effects of n-3 PUFA enrichment, differences in gene regulation, and differences in the animal diets. These species variations serve as a cautionary note about generalizing animal studies to the clinical situation. They also suggest that the effects of FO supplementation on the cardiac electrical activity in people can vary due to differences in genetic makeup and conditions of the heart.

## References

[1] London B, Albert C, Anderson ME, Giles WR, van Wagoner DR (2007). Omega-3 fatty acids and cardiac arrhythmias: prior studies and recommendations for future resarch: a report from the National Heart, Lung, and Blood Institute and Office of Dietary Supplements Omega-3 Fatty Acids and their Role in Cardiac Arrhythmogenesis Workshop.. Circulation.

[2] Marchioli R, Barzi F, Bomba E, Chieffo C, Di Gregorio D (2002). Early protection against sudden death by n-3 polyunsaturated fatty acids after myocardial infarction: time-course analysis of the results of the Gruppo Italiano per lo Studio della Sopravvivenza nell'Infarto Miocardico (GISSI)-Prevenzione.. Circulation.

[3] Xiao Y-F, Sigg DC, Leaf A (2005). The antiarrhythmic effect of n-3 polyunsaturated fatty acids: modulation of cardiac ion channels as a potential mechanism.. J Memb Biol.

[4] Xiao Y-F, Kang JX, Morgan JP, Leaf A (1995). Blocking effects of polyunsaturated fatty acids on Na^+^ channels of neonatal rat ventricular myocytes.. PNAS.

[5] Xiao Y-F, Gomez AM, Morgan JP, Lederer WJ, Leaf A (1997). Suppression of voltage-gated L-type Ca^2+^ currents by polyunsaturated fatty acids in adult and neonatal rat ventricular myocytes.. PNAS.

[6] Xiao Y-F, Ke Q, Wang S-Y, Auktor K, Yang Y (2001). Single point mutations affect fatty acid block of human myocardial sodium channel α subunit Na^+^ channels.. PNAS.

[7] Verkerk AO, van Ginneken ACG, Berecki G, Den Ruijter HM, Schumacher CA (2006). Incorporated sarcolemmal fish oil fatty acids shorten pig ventricular action potentials.. Cardiov Res.

[8] Folch J, Lees M, Sloanestanley GH (1957). A simple method for the isolation and purification of total lipids from animal tissues.. J Biol Chem.

[9] Christie WW, Christie WW (1989). Fatty acids and lipids: structures, extraction and fractionation into classes.. Gas Chromatography and Lipids.

[10] Jia Y, Takimoto K (2006). Mitogen-activated protein kinases control cardiac KChIP2 gene expression.. Circ Res.

[11] O'Rourke B, Kass DA, Tomaselli GF, Kaab S, Tunin R (1999). Mechanisms of altered excitation-contraction coupling in canine tachycardia-induced heart failure, I. Experimental studies.. Circ Res.

[12] Rose J, Armoundas AA, Tian Y, DiSilvestre D, Burysek M (2005). Molecular correlates of altered expression of potassium currents in failing rabbit myocardium.. Am J Physiol.

[13] Wickenden AD, Tsushima RG, Losito VA, Kaprielian R, Backx PH (1999). Effect of Cd^2+^ on Kv4.2 and Kv1.4 expressed in *Xenopus* oocytes and on the transient outward currents in rat and rabbit ventricular myocytes.. Cellular Physiology and Biochemistry.

[14] Bruno JJ, Koeppe REII, Andersen OS (2007). Docosahexaenoic acid alters bilayer elastic properties.. PNAS.

[15] Wassall SR, Brzustowicz MR, Shaikh SR, Cherezov V, Caffrey M (2004). Order from disorder, corralling cholesterol with chaotic lipids. The role of polyunsaturated lipids in membrane raft formation.. Chemistry and Physics of Lipids.

[16] Stulnig TM, Huber J, Leitinger N, Imre E-M, Angelisova P (2001). Polyunsaturated eicosapentaenoic acid displaces proteins from membrane rafts by altering raft lipid composition.. J Biol Chem.

[17] Pepe S (2005). Effect of dietary polyunsaturated fatty acids on age-related changes in cardiac mitochondrial membranes.. Experimental Gerontology.

[18] Edwards IJ, O'Flaherty JT (2008). Omega-3 fatty acids and PPARγ in cancer..

[19] Bogdanov KY, Spurgeon HA, Vinogradova TM, Lakatta EG (1998). Modulation of the transient outward current in adult rat ventricular myocytes by polyunsaturated fatty acids.. Am J Physiol.

[20] Dujardin KS, Dumotier B, David M, Guizy M, Valenzuela C (2008). Ultrafast sodium channel block by dietary fish oil prevents dofetilide-induced ventricular arrhythmias in rabbit hearts.. Am J Physiol.

